# Id3 and Bcl6 Promote the Development of Long-Term Immune Memory Induced by Tuberculosis Subunit Vaccine

**DOI:** 10.3390/vaccines9020126

**Published:** 2021-02-05

**Authors:** Jiangyuan Han, Yanlin Ma, Lan Ma, Daquan Tan, Hongxia Niu, Chunxiang Bai, Youjun Mi, Tao Xie, Wei Lv, Juan Wang, Bingdong Zhu

**Affiliations:** 1 Gansu Provincial Key Laboratory of Evidence Based Medicine and Clinical Translation & Lanzhou Center for Tuberculosis Research, School of Basic Medical Sciences, Lanzhou University, Lanzhou 730000, China; hanjy17@lzu.edu.cn (J.H.); myl19960701@163.com (Y.M.); hanjy14@lzu.edu.cn (L.M.); tandaquan@163.com (D.T.); niuhongxia1985@163.com (H.N.); baichx13@lzu.edu.cn (C.B.); miyoujun@126.com (Y.M.); xiet18@lzu.edu.cn (T.X.); lvw18@lzu.edu.cn (W.L.); wangj19@lzu.edu.cn (J.W.); 2 Institute of Pathogen Biology, School of Basic Medical Sciences, Lanzhou University, Lanzhou 730000, China; 3 Institute of Pathophysiology, School of Basic Medical Sciences, Lanzhou University, Lanzhou 730000, China

**Keywords:** IL-7, Bcl6, Id3, immune memory, vaccine

## Abstract

Long-lived memory cell formation and maintenance are usually regulated by cytokines and transcriptional factors. Adjuvant effects of IL-7 have been studied in the vaccines of influenza and other pathogens. However, few studies investigated the adjuvant effects of cytokines and transcriptional factors in prolonging the immune memory induced by a tuberculosis (TB) subunit vaccine. To address this research gap, mice were treated with the *Mycobacterium tuberculosis* (*M. tuberculosis*) subunit vaccine Mtb10.4-HspX (MH) plus ESAT6-Ag85B-MPT64_<190–198>_-Mtb8.4-Rv2626c (LT70), together with adeno-associated virus-mediated IL-7 or lentivirus-mediated transcriptional factor Id3, Bcl6, Bach2, and Blimp1 at 0, 2, and 4 weeks, respectively. Immune responses induced by the vaccine were examined at 25 weeks after last immunization. The results showed that adeno-associated virus-mediated IL-7 allowed the TB subunit vaccine to induce the formation of long-lived memory T cells. Meanwhile, IL-7 increased the expression of *Id3*, *Bcl6*, and *bach2*—the three key transcription factors for the generation of long-lived memory T cells. The adjuvant effects of transcriptional factors, together with TB fusion protein MH/LT70 vaccination, showed that both Bcl6 and Id3 increased the production of antigen-specific antibodies and long-lived memory T cells, characterized by high proliferative potential of antigen-specific CD4^+^ and CD8^+^ T cells, and IFN-γ secretion in CD4^+^ and CD8^+^ T cells, respectively, after re-exposure to the same antigen. Overall, our study suggests that IL-7 and transcriptional factors Id3 and Bcl6 help the TB subunit vaccine to induce long-term immune memory, which contributes to providing immune protection against *M. tuberculosis* infection.

## 1. Introduction

Tuberculosis (TB) is one of the global health problems mainly caused by *Mycobacterium tuberculosis* (*M. tuberculosis*) infection in humans. Bacillus Calmette–Guerin (BCG) is the only attenuated vaccine currently used to protect humans against TB, although its efficacy wanes in adults [1]. It is believed that BCG induces effector memory T cells (T_EM_) rather than central memory T cells (T_CM_), which leads to impaired protective efficiency of BCG in adults [2]. TB subunit vaccines such as LT70 [3], H56 [4], HyVac4 [5], and Ag85B-ESAT-6/CAF01 [6] can induce long-term protection against *M. tuberculosis*. However, there is still a need to investigate what factors affect the development of long-term immune protection induced by subunit vaccines so as to determine a way to improve the TB subunit vaccine-induced long-term immune memory, which would provide long-term protection against TB.

Cell-mediated immunity is regarded as a protective immune response against TB. Following antigen stimulation, naïve T cells will develop into effector T cells (T_eff_) with the CD62L^−^CD44^+^ phenotype, T_EM_ with the CD62L^−^CD44^+^ phenotype, and T_CM_ with the CD62L^+^CD44^+^ phenotype [7,8,9]. Memory T cells, particularly T_CM_, subsequently remain quiescent following pathogen clearance, and an immune response is exerted more quickly upon stimulation. The development of memory T cells is regulated by the cytokines and transcription factors [8,10,11]. Interleukin-7 (IL-7), which is mainly produced by epithelial and stromal cells, regulates host immunity by intervening in the development and differentiation of both T cells and B cells [12,13]. IL-7 binds to its receptor and subsequently activates the JAK-STAT pathway and modulates factors located in the nucleus to regulate the homeostasis of naïve T cells, memory T cells, and memory B cells [14,15]. IL-7 is crucial for T_CM_ survival [12], and regulates the generation of follicular helper T (Tfh) cells and the formation of B cells [16]. IL-7 has been administered as a vaccine adjuvant, which extends vaccine response and improves the long-term survival of CD8^+^ memory T cells [17].

Intranuclear transcriptional factors determine the development of memory T and B cells [11,18]. Several transcriptional factors, including Bcl6, Bach2, Id3, and Blimp1, have been described as key regulators for memory cell development. Among them, Bcl6 is essential for the differentiation of memory T cells and critical for the development of B cells in germinal centers (GCs) [18,19]. Bach2 also promotes a higher recall proliferation of memory CD4^+^ and CD8^+^ T cells [20,21]. Id3, an E-box-containing transcription suppressor, regulates the development of memory CD8^+^ T cells [21]. Id3 deficiency in CD8^+^ T cells impairs T_CM_ differentiation [22]. Blimp1 contributes to short-lived effector cells differentiation and is required to recall the response of memory T cells [23,24]. With regard to B cell development, Id3, Bcl6, and Bach2 are known to promote the processes of GC maintenance and memory B cell formation [25,26], while Blimp1 is required for plasma cell differentiation. Thus, distinct transcriptional programming directs memory cell generation and development following TB subunit vaccine immunizations.

In the current study, the adeno-associated virus-mediated IL-7 or lentivirus-mediated transcription factors Id3, Bcl6, Bach2, and Blimp1 were immunized, together with the TB fusion protein-based vaccine Mtb10.4-HspX (MH) [27] plus ESAT6-Ag85B-MPT64_(190–198)_-Mtb8.4-Rv2626c (LT70) [28] to investigate whether they can cause the TB subunit vaccine to induce long-term T cell-mediated and antibody-mediated immune responses.

## 2. Materials and Methods

### 2.1. Animals

In accordance with the Guide for the Care and Use of Laboratory Animals, female C57BL/6 mice aged 6–8 weeks were bred in a specific pathogen-free facility at the College of Traditional Chinese Medicine (Lanzhou, China). Animal experiments were conducted in strict conformance to the guidelines of the China Council on Animal Care and Use.

### 2.2. Subunit Vaccine and Single Antigen Preparation

The fusion protein MH and LT70 vaccines were prepared as previously described [28]. For one dose of vaccine (0.2 mL), 5 μg of MH and 5 μg of LT70 were mixed with adjuvant consisting of 250 μg N, N′-dimethyl-N, N’-dioctadecylammonium bromide (DDA) (Cat. No. A—29, Pioneer, China), and 50 μg of polyinosinic-polycytidylic acid [Poly (I:C)] (Cat. No. 30811-80-4, Kaiping Genuine, China). Single antigens of Ag85B, Rv2626C, and HspX were purified as described in a previous study [28,29] by using a Ni-NTA His column (Novagen).

### 2.3. Construction of Recombinant Adeno-Associated Virus Expressing IL-7

Recombinant adeno-associated viral (rAAV) vectors were provided by the Miaoling Company (Beijing Miaoling, China). Human IL-7 cDNA was synthesized by the Beijing Genomics Institute (China) and was then inserted into pAAV-MCS, resulting in pAAV-IL-7. The pAAV-green fluorescent protein (pAAV-EGFP)/pAAV-IL-7 (10 μg), AAV2-helper (15 μg), and pAAV-RC (10 μg) were mixed with Lipofectamine 3000 (Invitrogen, Grand Island, NY, USA) and transfected into AAV293 cells. The cell supernatant containing recombinant AAV was harvested and concentrated. The viral titer of rAAV was determined by real-time reverse transcription polymerase chain reaction (RT-PCR) and counting fluorescent dots inside infected HEK293 cells.

### 2.4. Construction of the Recombinant Lentivirus Encoding Transcriptional Factors

The recombinant lentiviral (rLV) vectors pMIG-EGFP, pMIG-Id3, pMIG-Bcl6, and pMIG-Bach2 were from the Jianzhu Chen Lab at the Massachusetts Institute of Technology [21]. The pMIG-Blimp1 vector was constructed with the pMIG-MCS vector by using the same method. The genes Id3, Bcl6, Bach2, and Blimp1 are all from mice. For the lentiviral packaging, HEK293T cells were transfected using Lipofectamine 3000 (Invitrogen, Grand Island, NY, USA) with 15 μg of helper plasmids, 10 μg of pCl-Eco, and 10 μg of recombinant plasmids pMIG-EGFP, pMIG-Id3, pMIG-Bcl6, pMIG-Bach2, or pMIG-Blimp1. The medium containing recombinant LV was harvested 24 and 48 h post-transfection. The viral titer was determined by counting fluorescent dots inside infected HEK293 cells with the gradient dilution method.

### 2.5. Vaccination

Mice were immunized with vaccine, vaccine + rAAV-EGFP, vaccine + rAAV-IL-7, vaccine + rLV-EGFP, vaccine + rLV-Id3, vaccine + rLV-Bcl6, vaccine + rLV-rBach2, or vaccine + rLV-Blimp1. Phosphate buffered solution (PBS) was used as a negative control and inoculated in mice with a total volume of 200 µL/dose subcutaneously once at 0 week. The recombinant adeno-associated virus and recombinant lentivirus were injected subcutaneously (*s.c.*) into the mice at a dose of 5 × 10^6^ PFU. The subunit vaccine was then administered *s.c.* at 200 μL/mouse. All the mice were immunized *s.c.* with the recombinant virus and subunit vaccine 3 times at 0, 2, and 4 weeks at intervals of 2 weeks. Serum was obtained from the mice on day 7 and the concentrations of interleukin-7 (IL-7) were detected by ELISA (Cat. No. 70-EK207/2-96, Hangzhou Multi science Biotech, Hangzhou, China).

### 2.6. Cell Sorting and qPCR

Mouse spleen lymphocytes immunized with the vaccine + rAAV-EGFP/rAAV-IL-7 were isolated 3, 5, and 7 days after the first immunization. Negative selection with magnetic beads was used to enrich CD8^+^ (Cat. No. 130-104-075, Miltenyi Biotech, Bergisch-Gladbach, Germany) and CD4^+^ T cells (Cat. No. 130-104-454, Miltenyi Biotech, Bergisch-Gladbach, Germany), whose RNA was extracted with TRIzol. Mice were immunized with vaccine plus rAAV-EGFP, rAAV-IL-7, rLV-EGFP, rLV-Id3, rLV-Bcl6, rLV-Bach2, or rLV-Blimp1, and the RNA was extracted from the lymph nodes and surrounding soft tissue on day 5. The cDNA was synthesized using a RevertAid First Strand cDNA Synthesis Kit (Thermo Fisher Scientific, Waltham, MA, USA) and gene expression profiling was conducted by RT-PCR using the StepOne Plus™ Real-Time PCR System (Applied Biosystems, ABI, Foster City, CA, USA). The relative mRNA expression was normalized by β-actin with the formula 2^−ΔΔCt^, where ΔCt = Ct^target gene^—Ct^gapdh^ and ΔΔCt = Ct^experiment group^—Ct^control group^. The primers were as follows:

(1) *gapdh* forward, 5′−AGTGGCAAAGTGGAGATT-3′; reverse, 5′-GTGGAGTCATACTGGAACA-3′;
(2) *Bcl6* forward, 5′−CGTGAGGTCGTGGAGAACAATA-3′; reverse, 5′-GATAAGAGGCTGGTGGTGTTGA-3′;
(3) *Bach2* forward, 5′−ACTGGTGTGCGAGAAGGAAAA-3′; reverse, 5′-GTATGAGGACAGGGCAGTAGC-3′;
(4) Blimp1 forward, 5′−GACAGAGGCCGAGTTTGAAGA-3′; reverse, 5′-GCGTGTTCCCTTCGGTATGTA-3′;
(5) *Id3* forward, 5′−CTCTTAGCCTCTTGGACGACAT-3′; reverse, 5′-CTGAAGGTCGAGGATGTAGTCT-3′;
(6) *IL-7* forward, 5′−CCACCCATGGCAAATTCCATGGCA-3′; reverse, 5′-TCTAGACGGCAGGTCAGGTCCAC-3′.

### 2.7. Splenocyte Proliferation Assay

5-Ethynyl-2′-deoxyuridine (EdU) incorporation was used to analyze the proliferation of memory T cells 25 weeks after the last immunization [3,30]. We isolated the lymphocytes (5 × 10^6^ cells/well) from spleens and incubated them with the following antigens: 5 μg/mL of HspX, 5 μg/mL of Rv2626C, and 5 μg/mL of Ag85B, for 7 d with 2 mL culture volume in 24-well plates. EdU (Click-iT™ EdU Flow Cytometry Assay Kit, Invitrogen™, OR, USA) was added on the third day, with half fresh serum-containing medium (30 μM/mL), and cultured until day 7. The collected cells were then treated according to the instructions recommended by the manufacturer of the Click-iT™ EdU Flow Cytometry Assay Kit. The cells were stained with anti-mouse monoclonal antibodies, including anti-CD8-PerCP (53-6.7, eBioscience, San Diego, CA, USA) and anti-CD4-PE (RM4-5, eBioscience, San Diego, CA, USA). A flow cytometry (ACEC Biosciences, Inc., Zhejiang, HangZhou, China) assay was performed to detect the EdU incorporation.

### 2.8. Intracellular Cytokine Staining

At 25 weeks after the last immunization, we detected the vaccine-induced re-expanded memory responses, as previously described [3]. First, the mice were stimulated with 5 × 10^6^ CFU BCG via intraperitoneal injection for 9 days before lymphocytes were isolated. During this period, the T_CM_ were supposed to be activated and developed into T_EM_ or effector T cells in vivo. Subsequently, the isolated lymphocytes were stimulated in vitro with the antigens HspX (5 μg/mL), Rv2626C (5 μg/mL), and Ag85B (5 μg/mL) for 4 h at 37 °C and 5% CO_2_ as the effector memory T cell developed into Teff and secreted cytokine IFN-γ. Meanwhile, BD GolgiPlug™ (containing Brefeldin A) was added and then incubated for 8 h at 37 °C and 5% CO_2_. These cells were collected by PBS and stained on the cell surface with anti-CD4-FITC (RM4-5) and anti-CD8-PerCP-Cy5.5 (53–6.7), as well as intracellularly stained with anti-IFN-γ-APC (XMG1.2, eBioscience) and anti-IL-2-PE (JES6-5H4, eBioscience) after they were fixed and permeabilized using the BD Cytofix/Cytoperm kit and then analyzed by flow cytometric assay (ACEC Biosciences, Inc., Zhejiang, HangZhou, China).

### 2.9. Detection of Ag85B-Specific Antibodies in Mouse Sera by ELISA

Ag85B-specific IgG, IgG1, and IgG2c in sera were detected by indirect ELISA 25 weeks after the last immunization. Firstly, 10 μg/well of Ag85B (in PBS solution) were added onto the plate at 4 °C overnight. Secondly, the double-diluted serum was added, and 100 μL of goat anti-mouse IgG (Solarbio, Beijing, China) and rabbit anti-mouse IgG1 and IgG2c (Rockland Immunochemicals Inc., Montgomery, PA, USA) conjugated with horseradish peroxidase were poured into each well at a dilution of 1:5000 and 1:12,000, respectively. The 3,3′,5,5′-tetramethylbenzidine (TMB) substrate was added at 200 μL/well and incubated at room temperature for 15 min. The reaction was then stopped by diluted sulfuric acid (1 mol/L) at 50 μL/well. The color was quantified at 450 nm. The serum in the PBS group was used as the negative control. The antibody titer was evaluated as a reciprocal of each endpoint dilution, and the average log value of all antibody titers was considered as the final result for each group.

### 2.10. Statistical Analysis

Data were statistically analyzed using GraphPad Prism 8 Software (GraphPad Software, San Diego, CA, USA). Statistical significance was determined by one-way ANOVA followed by a Tukey post hoc test. A *p*-value < 0.05 was considered statistically significant.

## 3. Results

### 3.1. IL-7 Enhanced the Immune Responses Induced by Long-Term Memory T Cells

The mice were given adeno-associated virus-mediated IL-7 (rAAV-IL-7), together with the TB subunit vaccine. The relative expression of *IL-7* in lymph nodes and surrounding soft tissue and the production of IL-7 in serum increased in the additional rAAV-IL-7 group compared to the vaccine plus rAAV-EGFP control or vaccine alone groups on day 5 and day 7, respectively (supplementary material Figure S1).

**Figure 1:**
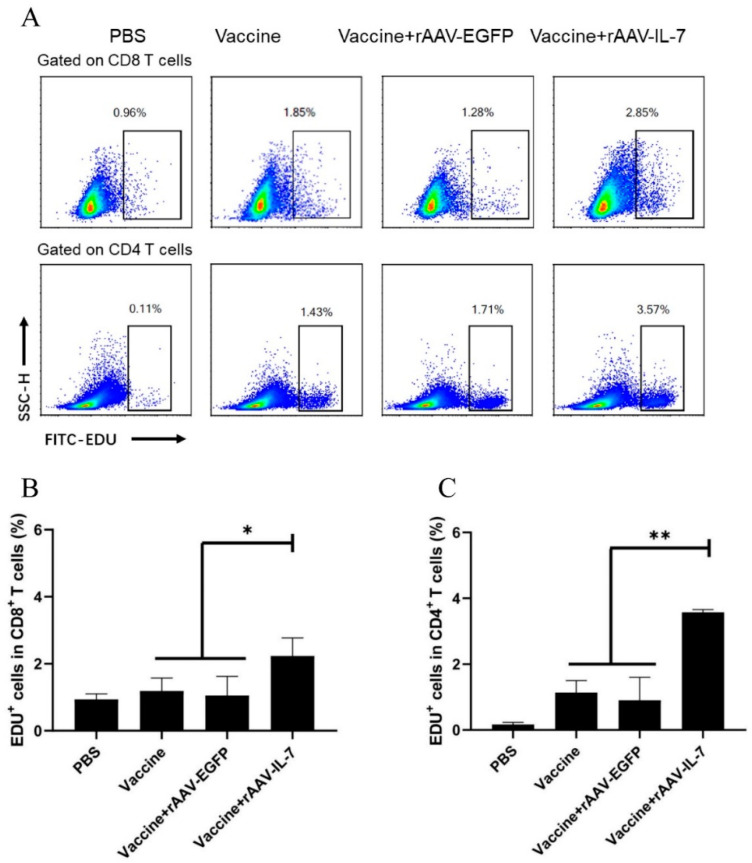
Vaccine plus adeno-associated virus-mediated IL-7 (rAAV-IL-7)-induced proliferation of antigen-specific T cells. Phosphate buffered solution (PBS) was used as a negative control. At 25 weeks after the last immunization, lymphocytes (5 × 10^6^ cells/well) were stimulated with mixed antigens HspX, Rv2626C, and Ag85B for 7 days, and 5-Ethynyl-2′-deoxyuridine (EdU) was added on day 3 to a final concentration of 30 μM. On day 7, the proliferative cells were determined by flow cytometry. (**A**) Flow cytometric analysis of the proliferation of CD8^+^ and CD4^+^ T cells. (**B**) Percentage of EdU^+^CD8^+^ T cells. (**C**) Percentage of EdU^+^CD4^+^ T cells. Each group consisted of 4 mice. * *p* < 0.05, ** *p* < 0.01.

To evaluate the generation of vaccine-induced antigen-specific memory T cells, T cell proliferation and cytokine production upon antigen stimulation were analyzed. At 25 weeks after the last immunization, the lymphocytes were isolated and then stimulated with mixed antigens for 7 days. EdU incorporation at day 3 was then analyzed at day 7. The results indicated that the proliferation of CD8^+^ and CD4^+^ T cells significantly increased in the vaccine combined with rAAV-IL-7 group (2.23 ± 0.54 and 3.57 ± 0.08 for CD8^+^ and CD4^+^ T cells, respectively) than the vaccine alone group (1.19 ± 0.38 and 1.13 ± 0.36 for CD8+ and CD4+ T cells, respectively) or vaccine plus rAAV-EGFP control group (1.05 ± 0.57 and 0.90 ± 0.69 for CD8^+^ and CD4^+^ T cells, respectively) following antigen stimulation (Figure 1A–C). This indicated that rAAV-IL-7 could help the vaccine to induce long-lived memory T cells which show a greater proliferative capacity.

**Figure 2:**
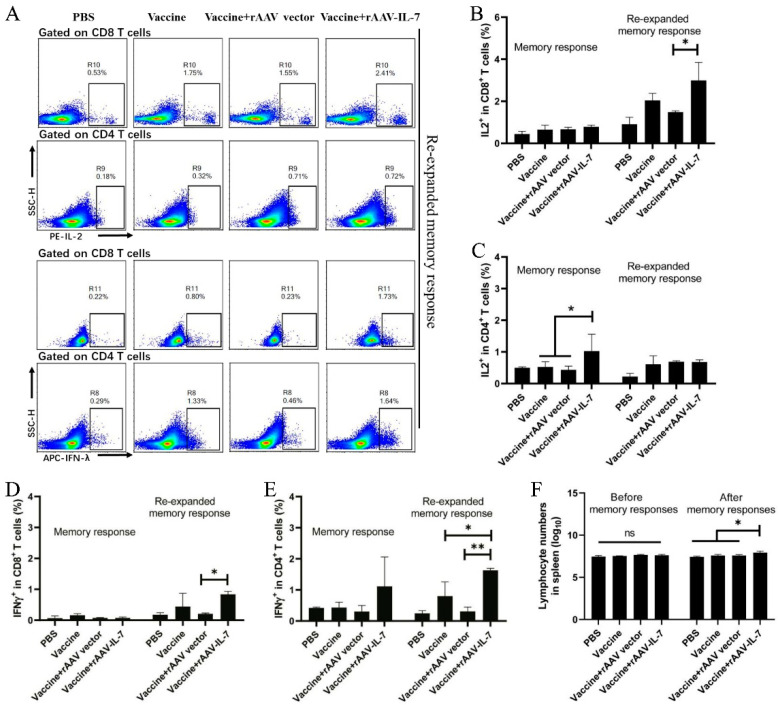
Vaccine plus rAAV-IL-7 induced the secretion of IL-2 and IFN-γ in T cells. At 25 weeks after the last immunization, the intracellular cytokine production by vaccine-induced T cells was analyzed. For memory responses, lymphocytes were stimulated with mixed antigens HspX, Rv2626C, and Ag85B in vitro. For re-expanded memory responses, the immunized mice were injected with Bacillus Calmette–Guerin (BCG) (1 × 10^6^ CFU) in vivo for 9 days and subsequently stimulated with mixed antigens HspX, Rv2626C, and Ag85B in vitro before intracellular cytokine staining assay by flow cytometry. (**A**) Flow cytometric analysis of intracellular IL-2 and IFN-γ produced by the spleen CD8^+^ and CD4^+^ T lymphocytes with BCG and antigen stimulation. (**B**) Frequencies of IL-2 produced by CD8^+^ T cells. (**C**) Frequencies of IL-2 produced by CD4^+^ T cells. (**D**) Frequencies of IFN-γ produced by CD8^+^ T cells. (**E**) Frequencies of IFN-γ produced by CD4^+^ T cells. (**F**) Lymphocyte counts before and after secondary immune response. Each group consisted of 4 mice. * *p* < 0.05, ** *p* < 0.01.

In addition, we detected the intracellular cytokine production 25 weeks after the last immunization. First, we determined the cytokine production following antigen stimulation within 12 h with the aim to detect the function of T_EM_. The results showed the frequency of IL-2-producing CD4^+^ T cells in the vaccine plus rAAV-IL-7 group (1.02 ± 0.53) increased compared to that of the vaccine alone group (0.52 ± 0.16) and the vaccine plus rAAV-EGFP group (0.43 ± 0.11) (Figure 2B,C, *p* < 0.05); meanwhile, no apparent difference in IFN-γ production was observed between different groups (Figure 2D,E). Second, to determine the immune responses of T_CM_, the mice given vaccines were intraperitoneally injected with BCG for 9 days before lymphocytes were isolated. The lymphocytes were stimulated with antigens for 12 h in vitro before cytokine determination. After BCG stimulation, the total spleen lymphocyte count in the additional rAAV-IL-7 group significantly increased compared to the other control groups (Figure 2F). The cytokine production of re-expanded memory responses was as follows: vaccine plus rAAV-IL-7 group exhibited significant increases in the frequencies of IL-2-producing CD8^+^ T cells (2.99 ± 0.85), IFN-γ-producing CD8^+^ T cells (0.84 ± 0.09), and IFN-γ-producing CD4^+^ T cells (1.06 ± 0.06), compared with that of the vaccine plus rAAV-EGFP group (Figure 2A–E). This difference could be attributed to the larger memory population in the IL-7 group than in the other groups. The results indicated that additional rAAV-IL-7, combined with the subunit vaccine, induced more CD8^+^ long-term memory T cells.

### 3.2. IL-7 Regulated the Expression of Transcription Factors Related to the Differentiation of Memory T Cells

**Figure 3:**
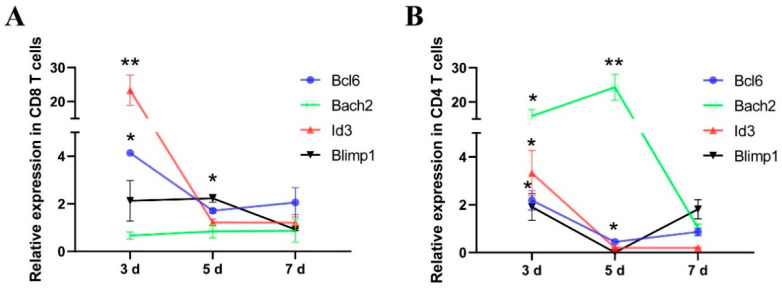
Vaccine plus rAAV-IL-7 regulated the expression of transcription factors in CD8^+^ and CD4^+^ T cells. Mice were immunized with the vaccine plus rAAV-green fluorescent protein (rAAV-EGFP) and the vaccine plus rAAV-IL-7. The expression levels of *Id3*, *Bcl6*, *Bach2*, and *Blimp1* in the CD8^+^ T cells (**A**) and CD4^+^ T cells (**B**) were determined on day 3, day 5, and day 7. Each group consisted of 4 mice. * *p* < 0.05, ** *p* < 0.01, relative to the vaccine plus rAAV-EGFP groups.

Following the immunization of the subunit vaccine and rAAV-IL-7, the expression levels of *Id3*, *Bcl6*, *Bach2*, and *Blimp1* were determined. In CD8^+^ T cells, the expression levels of *Bcl6* (4.13-fold) and *Id3* (23.31-fold) were significantly upregulated in 3 days, and the abundance of *Blimp1* (2.22-fold) significantly increased in 5 days with the additional rAAV-IL-7 treatment (Figure 3A). In CD4^+^ T cells, additional rAAV-IL-7 significantly upregulated *Id3* (3.32-fold), *Bcl6* (2.18-fold), and *Bach2* (15.86-fold) in 3 days (Figure 3B). This finding indicates that rAAV-IL-7 treatment combined with the subunit vaccine promoted the expression of *Id3*, *Bcl6*, and *Bach2* in the early stages of memory T cell formation.

### 3.3. Id3 and Bcl6 Enhanced the Proliferation of Memory T Cells

Mice were immunized with the TB subunit vaccine combined with LV-mediated transcription factors. The relative expression levels of *Id3*, *Bcl6*, *Bach2*, and *Blimp1* were detected on day 5 after vaccination. The expression levels of *Id3*, *Bcl6*, *Bach2*, and *Blimp1* all increased in the group of additional rLV-Id3, rLV-Bcl6, rLV-Bach2, and rLV-Blimp1 treatment groups, respectively, compared to the vaccine alone and vaccine plus rLV-EGFP groups (supplementary material Figure S1).

**Figure 4:**
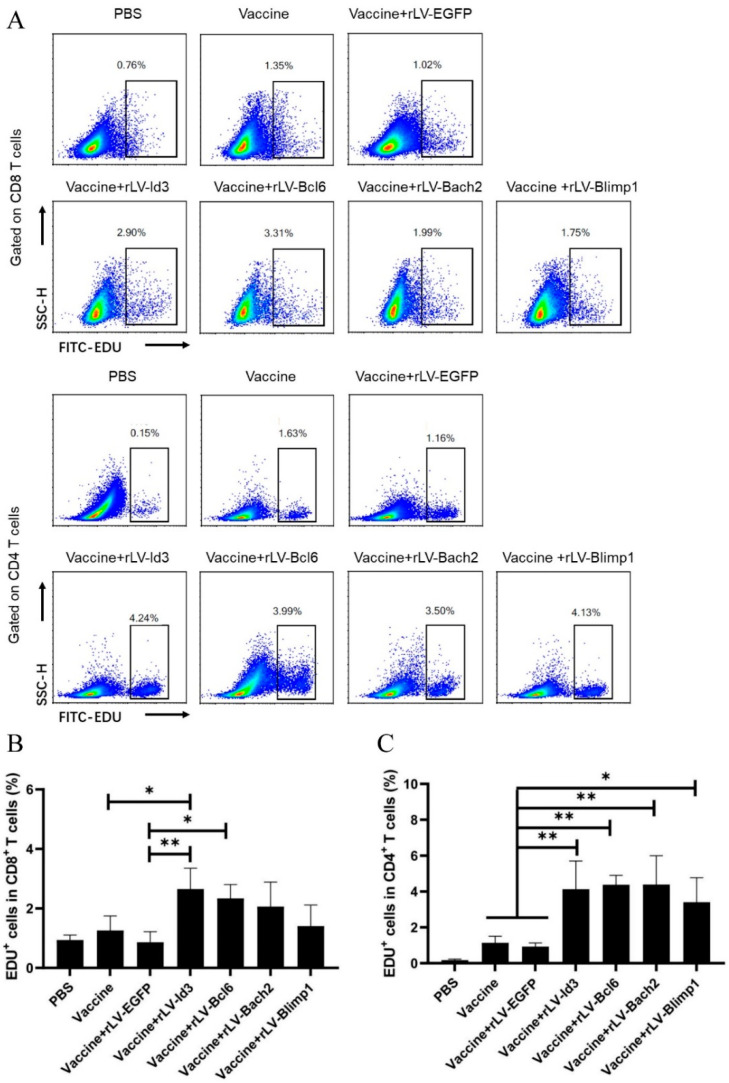
Lentivirus-mediated transcription factors affected antigen-specific T cell proliferation. At 25 weeks after the last immunization, lymphocytes (5 × 10^6^ cells/well) were stimulated with mixed antigens HspX, Rv2626C, and Ag85B for 7 days, and EdU was added on day 3 to a final concentration of 30 μM. On day 7, cell proliferation was determined by flow cytometry. (**A**) Flow cytometry of CD8^+^ and CD4^+^ T cell proliferation. (**B**) Percentage of EdU^+^CD8^+^ T cells. (**C**) Percentage of EdU^+^CD4^+^ T cells. Each group consisted of 4 mice. * *p* < 0.05, ** *p* < 0.01.

The proliferative capacity of the vaccine-induced T cells was analyzed by EdU staining 25 weeks after the last immunization. The results showed that the proliferation of antigen-specific CD8^+^ T cells was significantly enhanced in the additional rLV-Id3 treated group (2.65 ± 0.70), compared to the vaccine alone group (1.26 ± 0.48) and the vaccine plus control vector group (0.86 ± 0.355) (Figure 4A,B). Moreover, additional rLV-Bcl6 treatment increased the proliferation of CD8^+^ T cells (1.48 ± 0.28) (Figure 4A,B), which reflected the secondary recall capacity of the central memory population. The proliferation of antigen-specific CD4^+^ T cells increased in the vaccine plus rLV-Id3 (4.13 ± 1.57), rLV-Bcl6 (4.38 ± 0.52), rLV-Bach2 (4.39 ± 1.60), and rLV-Blimp1 (3.40 ± 1.36) groups, compared to the vaccine alone (1.13 ± 0.36) and vaccine plus rLV-EGFP groups (0.93 ± 0.19) (Figure 4A,C). Taken together, the transcription factors rLV-Id3 and rLV-Bcl6 significantly enhanced the proliferative capacity of CD4^+^ and CD8^+^ T cells following the TB subunit vaccine immunizations.

### 3.4. Bcl6 Increased IFN-γ Production in CD8^+^ T Cells, and Id3 Improved IFN-γ Production in CD4^+^ T Cells

**Figure 5:**
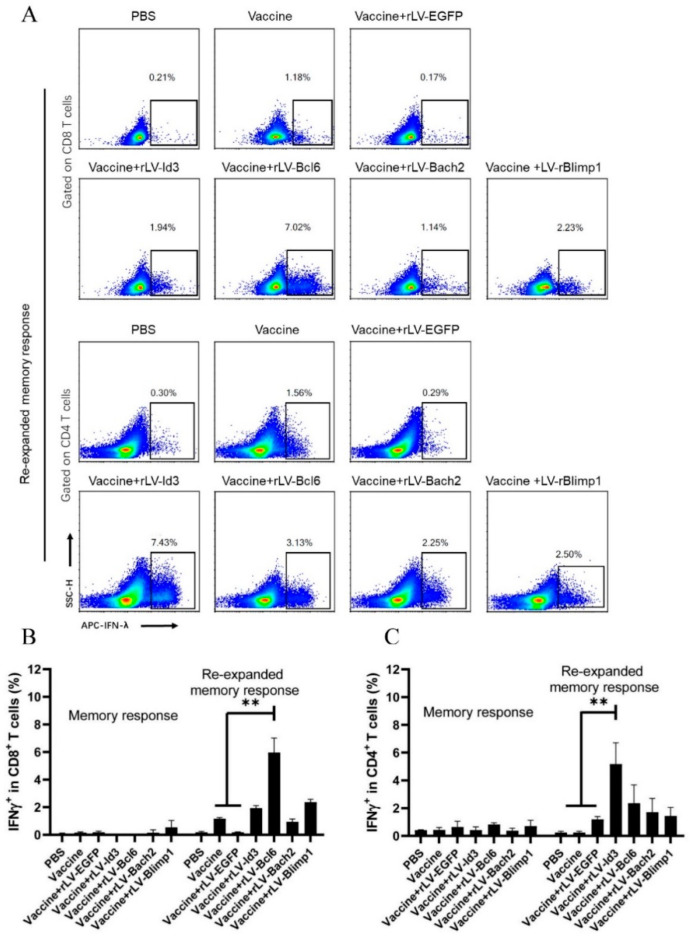
Lentivirus-mediated transcription factors affected IFN-γ secretion in CD8^+^ and CD4^+^ T cells. The immunized mice were injected with BCG (1 × 10^6^ CFU) in vivo for 9 days and subsequently stimulated with mixed antigens HspX, Rv2626C, and Ag85B in vitro before intracellular cytokine staining assay, which was determined by flow cytometry. (**A**) Flow cytometric analysis of intracellular IFN-γ produced by spleen CD8^+^ and CD4^+^ T lymphocytes after BCG and antigen stimulation. (**B**) Frequencies of IFN-γ-secreting CD8^+^ T cells; (**C**) Frequencies of IFN-γ-secreting CD4^+^ T cells. Each group consisted of 4 mice. ** *p* < 0.01.

To examine the cytokine production of vaccine-induced T_CM_, we analyzed IFN-γ secretion by CD8^+^ and CD4^+^ T cells from the mice treated with lentivirus-mediated transcription factors, together with subunit vaccine immunization 25 weeks after the last immunization. No apparent IFN-γ production was observed after special antigen stimulation for 12 h in vitro, suggesting that the vaccine-induced T_EM_ had waned 25 weeks after the last immunization. However, after BCG stimulation in vivo and special antigen stimulation in vitro, CD8^+^ T cells in mice from the vaccine plus rLV-Bcl6 group produced more IFN-γ (5.96 ± 1.04), compared with those in the vaccine alone (1.16 ± 0.08) and vaccine plus rLV-EGFP groups (0.18 ± 0.03) (Figure 5A,B). Meanwhile, additional rLV-Id3 (5.17 ± 1.54) enhanced the frequency of IFN-γ-secreting CD4^+^ T cells after BCG and antigen stimulation (Figure 5A,C, *p* < 0.01). This finding indicates that overexpression of Bcl6 and Id3 enhanced the formation of long-term memory CD8^+^ and CD4^+^ T cells, respectively.

### 3.5. Id3 and Bcl6 Enhanced the Production of Ag85B-Specific Antibodies

The production of Ag85B-specific antibodies in sera was determined 25 weeks after the last immunization. As shown in Table 1, the vaccine plus rAAV-IL-7 significantly increased the titers of IgG against Ag85B compared to the vector control (*p* < 0.05). As for the transcription factors, compared to the rLV-EGFP control, rLV-Id3 significantly increased the production of Ag85B-specific IgG and IgG2c (*p* < 0.05), and rLV-Bcl6 promoted the production of IgG1 (*p* < 0.05), while rLV-Bach2 decreased the Ag85B-specific IgG1 (*p* < 0.05).

## 4. Discussion

In this study, we confirmed that IL-7 helped the TB subunit vaccine induce long-term memory T cells by enhancing the proliferation of both CD4^+^ and CD8^+^ T cells, promoting more IL-2 production in CD4^+^ T cells upon antigenic stimulation, and increasing the production of IFN-γ production in both CD4^+^ and CD8^+^ T cells after successive BCG and antigen stimulation in vivo and in vitro. Meanwhile, we found that IL-7 increased the expression of *Id3*, *Bcl6*, and *bach2* in CD4^+^ and CD8^+^ T cells. We also evaluated the adjuvant effects of these transcription factors on vaccines. The results showed that the lentivirus-mediated transcription factors Id3 and Bcl6 enhanced the proliferation of both CD4^+^ and CD8^+^ T cells and the antibody production induced by the TB subunit vaccine. Moreover, we found that additional rLV-Id3 further induced long-term CD4^+^ memory T cells, whereas additional rLV-Bcl6 mainly helped induce more long-term CD8^+^ memory T cells.

Memory T cells are classified as T_CM_ and T_EM_ [9]. Memory T cells, particularly T_CM_, exhibit the capacity for differentiation, proliferation, and self-renewal when previous antigens are re-encountered [31,32,33]. In this experiment, we detected immune responses 25 weeks after the last immunization, when the effector T cells and most T_EM_ had waned. Therefore, the cell-mediated immune responses were mainly caused by T_CM_. To analyze the immune responses of vaccine-induced T_CM_, we evaluated re-expanded memory responses, in which T_CM_ proliferated and differentiated into T_EM_ upon antigen stimulation. Consequently, T_EM_ would generate the cytokine IFN-γ upon re-exposure to the same antigen. We stimulated the vaccine-immunized mice with BCG for 9 days in vivo and then the isolated lymphocytes were stimulated with special antigens in vitro, and the re-expanded memory responses were determined [3,34,35].

We observed that IL-7 had helped the TB subunit vaccine to induce the formation of long-term memory T cells, which exhibited improved re-expanded memory responses and increased proliferative capacity [34,36]. First, IL-7 treatment enhanced the proliferation of T cells and the production of IL-2 and IFN-γ with antigen stimulation. It was reported that IL-7 could control the proliferation and survival of CD4^+^ memory T cells [37]. In addition, the IL-7 treatment during the contraction phase of CD8^+^ T cells promoted the proliferation of CD8^+^ memory T cells and expanded the memory CD8^+^ T cell pool [38]. These experiments indicated that IL-7 could enhance vaccine-induced memory responses.

Transcription factors are among the key regulators for the generation of memory T cells [11,39]. In the current study, IL-7 activated the expression of *Id3*, *Bcl6*, and *Bach2*, suggesting that IL-7-induced transcription factors could promote T cell differentiation. Moreover, lentivirus-mediated Id3 and Bcl6 promoted the generation of long-lived memory T cells, enhanced the proliferation of vaccine-induced memory T cells, and increased the IFN-γ secretion in CD4^+^ T cells and CD8^+^ T cells, after antigen stimulation. This suggests that Id3 and Bcl6 help the TB subunit vaccine to generate long-lived memory CD4^+^ and CD8^+^ T cells, respectively. Id3 reportedly promoted the formation of memory CD8^+^ T cells upon infection [22,39]. The current study indicates that Id3 enhanced the formation of vaccine-induced memory T cells, particularly CD4^+^ T cells. Bcl6 could enhance the proliferative capacity of memory CD8^+^ T cells [40]. Deletion of Bcl6 in CD4^+^ T cells could abrogate the generation of T_CM_ [41]. Those findings were consistent with the results of the current study that Bcl6 could help the TB subunit vaccine induce long-term memory T cells, particularly CD8^+^ T cells. This strongly suggests that a combination of Id3 and Bcl6 with the vaccine could induce long-lived memory T cells and produce a long-term immune response. Bach2 was required for the memory T cell differentiation, and the deficiency of Bach2 severely impaired the generation of memory CD4^+^ T cells [20,42]. We also found that Bach2 promoted the proliferation of CD4^+^ T cells; however, IFN-γ secretion in the re-expanded memory response showed no increase. The effect of Bach2 regulation on immune memory has yet to be clarified.

Transcription factors were vital for the differentiation of memory B cells [18,43,44]. The present study showed that higher Ag85B-specific IgG and IgG2c levels were induced by overexpressed Id3, while the overexpressed Bcl6 mainly increased the Ag85B-specific IgG1. These finding were consistent with the results on the proliferation of CD4^+^ T cells. The development of memory B cells required the presentation of antigens to CD4^+^ memory Tfh cells [43]. In addition, Bcl6 could maintain the long-term survival of memory B cells by suppressing Blimp1 expression [18,43]. Id3 was essential for the development of B cells by suppressing E2A in early pre-B cells [25]. The current study showed that Id3 and Bcl6 could help the subunit vaccine to induce the formation of the long-term memory B cells.

## 5. Conclusions

In summary, IL-7 increased the expression of transcriptional factors Id3 and Bcl6 in CD4^+^ and CD8^+^ T cells in the process of subunit vaccine immunization. IL-7 and transcription factors Id3 and Bcl6 could promote the generation of TB subunit vaccine-induced long-lived memory T cells and long-term antibody production. Our previous research found that the quality and quantity of T_CM_ were related to the protection against *Mycobacterium* BCG challenge [3]. Other laboratories also demonstrated that the generation of T_CM_ promoted protective efficacy against *M. tuberculosis* challenge [45]. Therefore, IL-7 and transcription factors Id3 and Bcl6 could help the TB subunit vaccine to induce long-term immune memory, which would provide long-term immune protection against *M. tuberculosis* infection.

## Figures and Tables

**Table 1:** The production of Ag85B-specific IgG, IgG1, and IgG2c.

Groups	Antibody Titers			
	IgG	IgG1	IgG2c	IgG2c/IgG1
Vaccine	3.31 ± 0.59	2.37 ± 0.12	2.19 ± 0.48	0.92 ± 0.20
Vaccine + rAAV-EGFP	3.27 ± 0.15	2.33 ± 0.36	2.48 ± 0.92	1.06 ± 0.07
Vaccine + rAAV-IL-7	3.58 ± 0.03 *^,#^	3.37 ± 0.16	2.84 ± 0.10	0.84 ± 0.03
Vaccine + rLV-EGFP	3.31 ± 0.53	2.70 ± 0.13	2.40 ± 0.24	0.89 ± 0.09
Vaccine + rLV-Id3	3.62 ± 0.11 *^,$^	3.01 ± 0.13 *	3.10 ± 0.00 *^$^	1.02 ± 0.00
Vaccine + rLV-Bcl6	3.43 ± 0.13	3.23 ± 0.15 *^,$^	2.98 ± 0.12 *	0.92 ± 0.04
Vaccine + rLV-Bach2	3.47 ± 0.05	2.11 ± 0.18 ^$^	2.93 ± 0.07 *	1.38 ± 0.19 ^$^
Vaccine + rLV-Blimp1	3.03 ± 0.02	2.95 ± 0.15	2.72 ± 0.20	0.92 ± 0.07

Data are expressed as means ± standard deviation (SD) (*n* = 4). Antibody titers are expressed as the reciprocal end point titers, and the data are presented as the means of log10 antibody titers ± SD. * *p* < 0.05 *vs*. vaccine; ^#^ *p* < 0.05 *vs*. vaccine plus rAAV-EGFP; and ^$^ *p* < 0.05 *vs*. vaccine plus or rLV-EGFP.

## Data Availability

The data presented in this study are available on request from the corresponding author.

## Supplementary Materials

The following are available online at https://www.mdpi.com/2076-393X/9/2/126/s1, Figure S1: Expression of IL-7, *Id3*, *Bcl6*, *Bach2*, and Blimp1 in lymph nodes and surrounding soft tissue and the IL-7 production in serum.

## Abbreviations

TB	Tuberculosis
*M. tuberculosis*	*Mycobacterium tuberculosis*
BCG	*Mycobacterium bovis* Bacillus Calmette–Guerin
T_eff_	effector T cells
T_CM_	central memory T cells
T_EM_	effector memory T cells
JAK-STAT pathway	Janus kinase signal transducer and activator of transcription pathway
Tfh	follicular helper T cell
GCs	germinal centers
DDA	dioctadecylammonium bromide
Poly (I:C)PBS	polyinosinic-polycytidylic acidphosphate buffered solution
TMB	3,3′,5,5′-tetramethylbenzidine
EdU	5-Ethynyl-2′-deoxyuridine
IL	interleukin
LT70	ESAT6-Ag85B-MPT64<190-198>-Mtb8.4-Rv2626c
MH	Mtb10.4-HspX
H56	Ag85B-ESAT-6-Rv2660c
HyVac4	Ag85B-TB10.4
HEK	human embryonic kidney
PBS	phosphate-buffered saline
rLV	recombined lentivirus
rAAV	recombined adeno-associated virus
*s.c.*	subcutaneously
E2A	E-protein family transcription factor
