# Supplementary material for: Id3 and Bcl6 Promote the Development of Long-Term Immune Memory Induced by Tuberculosis Subunit Vaccine
Source: Vaccines (Basel). 2021 Feb 5;9(2):126. doi: 10.3390/vaccines9020126 (PMC7914852; doi:10.3390/vaccines9020126)
Supplement: Supplementary file 1 [file vaccines-09-00126-s001.pdf]

vaccines

MDPI

Supplementary information:

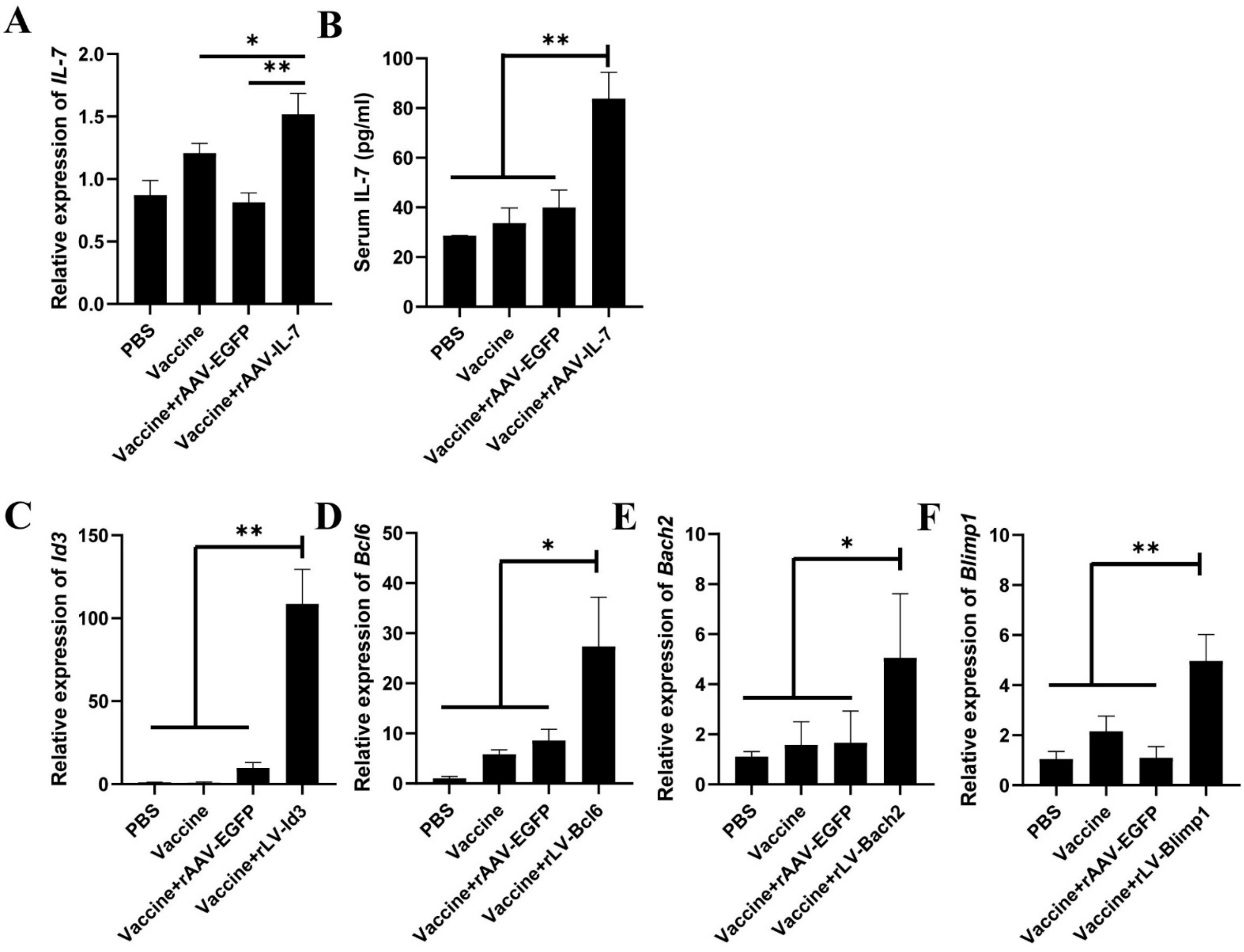

Figure S1. Expression of *IL-7*, *Id3*, *Bcl6*, *Bach2* and *Blimp1* in lymph nodes and surrounding soft tissue and the production of IL-7 in serum. Mice were immunized with the vaccine plus rAAV-EGFP, rAAV-IL-7 or rLV-EGFP, rLV-Id3, rLV-Bcl6, rLV-Bach2, and rLV-Blimp1. The relative expression of *IL-7*, *Id3*, *Bcl6*, *Bach2*, and *Blimp1* was determined on day 5. IL-7 production in the serum with the additional rAAV-EGFP/rAAV-IL-7 was detected on day 7. Each group consisted of 3 mice. *$p < 0.05$, **$p < 0.01$.
